# Are Sarcopenia and Myosteatosis in Elderly Patients with Pelvic Ring Injury Related to Mortality, Physical Functioning and Quality of Life?

**DOI:** 10.3390/jcm10214874

**Published:** 2021-10-22

**Authors:** Hester Banierink, Julia J. C. Bombach, Kaj ten Duis, Frank F. A. IJpma, Erik Heineman, Sven H. van Helden, Robert J. Nijveldt, Alain R. Viddeleer, Inge H. F. Reininga

**Affiliations:** 1 Department of Trauma Surgery, University Medical Center Groningen, University of Groningen, 9713 GZ Groningen, The Netherlands; j.j.c.bombach@umcg.nl (J.J.C.B.); k.ten.duis@umcg.nl (K.t.D.); f.f.a.ijpma@umcg.nl (F.F.A.I.); i.h.f.reininga@umcg.nl (I.H.F.R.); 2 Department of Surgery, University Medical Center Groningen, University of Groningen, 9713 GZ Groningen, The Netherlands; e.heineman@umcg.nl; 3 Department of Surgery, Isala Hospital Zwolle, 8025 AB Zwolle, The Netherlands; s.h.van.helden@isala.nl (S.H.v.H.); r.j.nijveldt@isala.nl (R.J.N.); 4 Medical Imaging Center, Department of Radiology, University Medical Center Groningen, University of Groningen, 9713 GZ Groningen, The Netherlands; a.r.viddeleer@umcg.nl

**Keywords:** pelvic ring injury, sarcopenia, myosteatosis, survival, quality of life, physical functioning

## Abstract

The purpose of this study was to evaluate the prevalence of sarcopenia and/or myosteatosis in elderly patients with pelvic ring injuries and their influence on mortality, patient-perceived physical functioning and quality of life (QoL). A multicenter retrospective cohort study was conducted including elderly patients aged ≥ 65 treated for a pelvic ring injury. Cross-sectional computed tomography (CT) muscle measurements were obtained to determine the presence of sarcopenia and/or myosteatosis. Kaplan–Meier analysis was used for survival analysis, and Cox proportional hazards regression analysis was used to determine risk factors for mortality. Patient-reported outcome measures for physical functioning (SMFA) and QoL (EQ-5D) were used. Multivariable linear regression analyses were used to determine the effect of sarcopenia and myosteatosis on patient-perceived physical functioning and QoL. Data to determine sarcopenia and myosteatosis were available for 199 patients, with a mean follow-up of 2.4 ± 2.2 years: 66 patients (33%) were diagnosed with sarcopenia and 65 (32%) with myosteatosis, while 30 of them (15%) had both. Mortality rates in patients at 1 and 3 years without sarcopenia and myosteatosis were 13% and 21%, compared to 11% and 36% in patients with sarcopenia, 17% and 31% in patients with myosteatosis and 27% and 43% in patients with both. Higher age at the time of injury and a higher Charlson Comorbidity Index (CCI) were independent risk factors for mortality. Patient-reported mental and emotional problems were significantly increased in patients with sarcopenia.

## 1. Introduction

Pelvic ring injuries in frail elderly patients are a growing health concern as the population ages. One third of all injuries and 73% of all pelvic ring injuries occur in the elderly [1]. Changes in body composition take place with age. Frailty, known as aging-related physiological decline, is characterized by vulnerability to adverse health outcomes. A surrogate measure of frailty is the gradual decline in skeletal muscle mass and strength (sarcopenia), which can act synergistically with an increase in intermuscular and intramuscular fat (myosteatosis). The exact mechanisms of sarcopenia and myosteatosis are still unknown, but both have been associated with aging and inactivity. It is estimated that up to 25% of persons under age 70 and over 50% of those 80 or older have sarcopenia [2]. Due to the rapidly expanding aging population, it is roughly estimated that sarcopenia will affect over 200 million people worldwide in the next 30 years [3].

Numerous studies have described the harmful health effects of sarcopenia and myosteatosis. Sarcopenia increases the likelihood of falls and injuries [4,5] and could therefore be considered a potential complementary predictive value for fracture risk [6]. Sarcopenia is also associated with increased rates of osteoporosis, morbidity and mortality [6,7,8]. Pelvic ring injuries are likewise known for their high mortality rates, which are estimated at 15% [9]. In the elderly, mortality can even rise up to 27% at 1 year [10]. It has been shown that patients suffering from pelvic ring injuries deal with decreased patient-reported physical functioning and quality of life (QoL) [10,11]. Sarcopenia and myosteatosis are seen as important determinants of physical functioning and QoL. The loss of skeletal muscle mass directly contributes to exercise intolerance, impaired ability to perform activities of daily living and loss of independence [3,5,12]. Still, the use of sarcopenia and myosteatosis as measures for frailty in musculoskeletal-related literature is sparse and little is known about the prevalence, mortality and effect on patient-perceived physical functioning and QoL in patients with pelvic ring injuries.

The aim of this study was to assess the prevalence of sarcopenia and myosteatosis in patients with pelvic ring injuries. We subsequently evaluated the association between the presence of sarcopenia and/or myosteatosis in patients with pelvic ring injuries and mortality, physical functioning and QoL.

## 2. Materials and Methods

### 2.1. Patients

A retrospective cohort study was conducted including all patients aged 65 or older and treated for a pelvic ring injury at the trauma surgery departments of two level-1 trauma centers in the Netherlands between 2007 and 2020 (UMCG Groningen and Isala Hospital Zwolle). Inclusion criteria were patients aged ≥ 65, a CT scan at the time of injury including the fourth lumbar vertebra (L4) and available data on patients’ height. Exclusion criteria were patients unable to read Dutch, severe mental disabilities and traumatic brain injury with neurological symptoms. The UMCG Medical Ethics Review Board assessed the methods employed and waived further need for approval (METc 2016.385 and METc 2017.543).

### 2.2. Data Acquisition

Data of patients treated for a pelvic ring injury between 2007 and 2016 were gathered retrospectively, while from 2017 onwards, data were collected prospectively. Demographic data and information related to the injury and treatment were extracted from patients’ medical and surgical records. Body mass index (BMI) classification was based on the World Health Organization (WHO) definitions [13]: for adults, overweight is defined as BMI ≥ 25 and obesity as BMI ≥ 30. Injury mechanisms were divided into low-energy trauma or high-energy trauma. Low-energy trauma is defined as a fall below two to three times the body height. High-energy trauma can be a fall above two to three times the body height, compression injuries, crush injuries or injuries from traffic accidents [14]. The Injury Severity Score (ISS) [15] was retrieved from the Dutch Trauma Registry. The ISS provides information about mortality, morbidity and other measures of severity, and can range from 1 to 75. An ISS score ≥ 16 indicates that a patient is severely injured. Subsequently, two trauma surgeons with ample experience in pelvic injury surgery reassessed the radiographic images (plain anteroposterior, inlet and outlet radiographs and CT scans) of all the patients and classified the pelvic ring injuries into type A, B and C injuries according to the AO/OTA trauma pelvis manual [16]. Operative treatment consisted of anatomical reduction and fixation of the pelvis. Non-operative treatment of pelvic ring injuries consisted of early mobilization with weight-bearing as tolerated in combination with appropriate pain medication. The patient’s comorbid conditions were classified according to the Charlson Comorbidity Index (CCI) [17]. Complications that occurred within 30 days were extracted from the medical charts and reviewed.

### 2.3. Muscle Imaging

**Figure 1:**
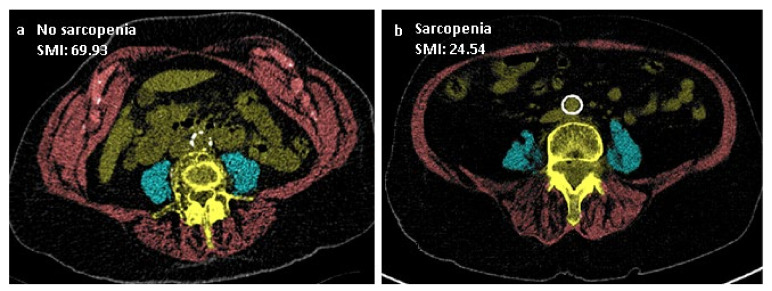
Cross-sectional muscle measurement at the level of the fourth lumbar vertebra (**a**,**b**). The blue area identifies the psoas major muscle. The red area represents the abdominal wall and the erector spinae muscles. Together, they are used to form the skeletal muscle index (SMI), calculated as (muscle area)/(patient height)^2^.

**Figure 2:**
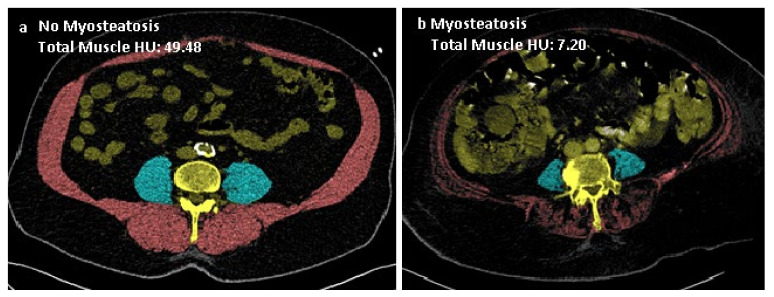
Cross-sectional muscle measurement at the level of the fourth lumbar vertebra (**a**,**b**). The blue area identifies the psoas major muscle. The red area represents the abdominal wall and the erector spinae muscles. Total muscle Hounsfield units (HU) were calculated to define myosteatosis.

CT imaging was performed on all patients shortly after arrival at the hospital on a Siemens SOMATOM Definition (AS, Edge, Flash), Force or Sensation (Siemens Medical, Erlangen, Germany) scanner. Slice thickness varied between 0.5 and 5 mm. CT slices were acquired with a 512 × 512 matrix and, after anonymization, stored in DICOM format for further processing. All CT scans were reassessed, and the CT slice, at the level of L4 where both transverse processes were best shown, was selected for each patient. Cross-sectional muscle measurements were obtained at this level. Image analysis was performed blinded by a radiologist with ample experience. The muscles assessed for measurements of sarcopenia and myosteatosis consisted of the psoas major and abdominal wall, including the erector spinae, quadratus lumborum, transversus abdominis, obliquus internus, obliquus externus and rectus abdominis (Figure 1 and Figure 2). In-house developed software (SarcoMeas 0.34; UMCG, Groningen, The Netherlands) was used to assess skeletal muscle mass, in order to determine the presence of sarcopenia and myosteatosis (Figure 1 and Figure 2). This software allows for manual delineation of the area of interest with semiautomatic assessment of skeletal muscle area based on tissue attenuation. According to the standard of Mitsiopoulos et al., muscle voxels were defined within the drawn contours by selecting all voxels with a radiodensity between −29 and +150 Hounsfield units (HU) [18]. The obtained cross-sectional skeletal muscle area was subsequently normalized with respect to squared body height to form the skeletal muscle index (SMI), calculated as (muscle area)/(patient height)^2^. The SMI is used as an index for sarcopenia. Mean radiodensity of all muscle voxels was calculated to assess myosteatosis.

### 2.4. Evaluating Physical Functioning and Quality of Life

Patients alive at follow-up were approached and asked to complete a series of patient-reported outcome measures to assess long-term physical functioning and QoL. Patients from the retrospective cohort received these questionnaires at a single moment in 2017 after at least a one-year follow-up. Patients from the prospective cohort received these questionnaires one year after the injury.

Physical functioning was measured with the Dutch version of the Short Musculoskeletal Function Assessment (SMFA-NL) [19]. The SMFA contains 46 items that are scored on a 5-item Likert scale. Two indices (function and bother) [20] and four subscales (upper extremity dysfunction, lower extremity dysfunction, problems with daily activities, mental and emotional problems) can be calculated [19]. Scores are calculated by summing up the individual items and transforming scores in a range from 0 to 100, with higher scores indicating better function. The SMFA-NL has been shown to be a valid and reliable questionnaire in injured patients [19,21].

QoL was measured with the EuroQol-5D (EQ-5D). The EQ-5D is a questionnaire that measures health-related QoL and consists of five items: mobility, self-care, daily activities, pain/discomfort and anxiety/depression [22], scored on a 5-item Likert scale. Based on these values, a utility score ranging from 0 to 1 was formed, with higher scores indicating better function. The EQ-5D has been shown to be a valid and reliable questionnaire in injured patients [23].

### 2.5. Statistical Analysis

Descriptive statistics were performed to present demographics, injury patterns and treatment. Means and standard deviations were calculated from normally distributed data and the median and interquartile range from non-parametric data. Based on the SMI and radiodensity of the total musculature, patients were divided as having no sarcopenia/no myosteatosis, sarcopenia/no myosteatosis, myosteatosis/no sarcopenia and both sarcopenia and myosteatosis. Sex-specific SMI were determined, with the lower tertile splits defining sarcopenia (low SMI). BMI-specific (<25 and ≥25) cut-off values were used for HU, with the lower tertile splits defining myosteatosis (low HU). Either independent samples *t*-test or Mann–Whitney U-test were performed to assess differences between groups. Categorical variables were evaluated by using the Chi-squared test. Kaplan–Meier survival analysis was used to assess long-term survival, and Cox proportional hazards regression analysis was used to evaluate whether sex, age (65–75, 76–85, >85), sarcopenia and/or myosteatosis, as categorized above, CCI (2–3 vs. ≥4) and ISS (<16 or ≥16) were predictive factors for mortality. Non-response analyses were performed to evaluate differences between (1) patients with and without sarcopenia and myosteatosis measurements, and (2) patients who responded to the questionnaires and those who did not. Multivariable linear regression analyses were performed to evaluate the association between physical functioning, QoL and sarcopenia and myosteatosis, corrected for CCI, BMI and age as possible confounders. A subset of the data was analyzed separately that only included scans without an intravenous contrast agent, as this may influence HU and thus myosteatosis measurements [24]. A *p*-value < 0.05 was considered to indicate statistical significance. All statistical analyses were performed using IBM SPSS software, v. 23.

## 3. Results

### 3.1. Demographics

**Figure 3:**
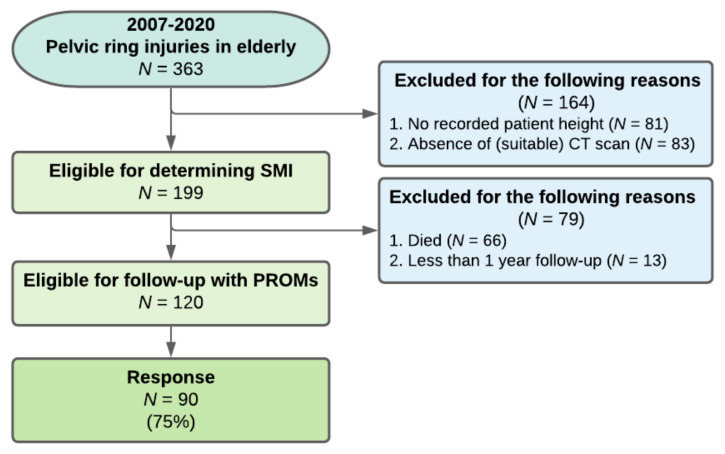
Flowchart of patient inclusion for assessment of skeletal muscle index, long-term physical functioning and quality of life after pelvic ring injuries at follow-up. The thirteen patients with less than a one-year follow-up were included in the survival analysis but excluded from the PROMs assessment.

A total of 363 patients (aged ≥ 65) with a pelvic ring injury were identified over a study period of 14 years (January 2007 to January 2021) (Figure 3).

**Figure 4:**
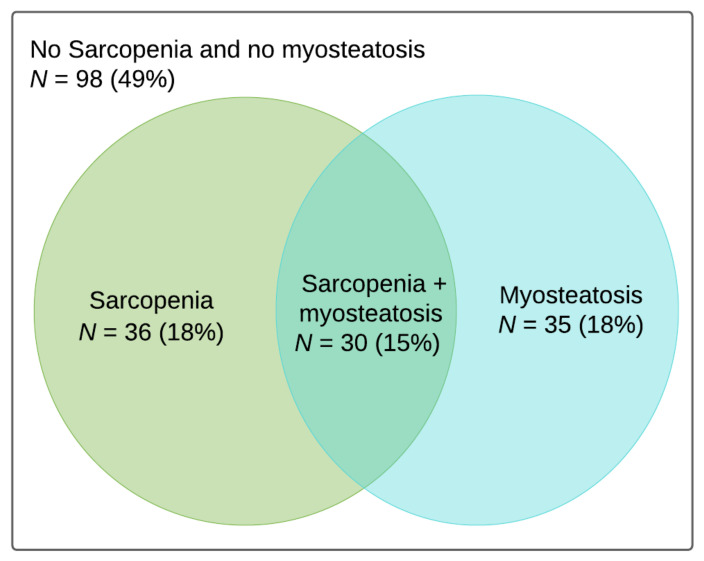
Venn diagram showing patients with sarcopenia, myosteatosis, both or neither.

For 199 (55%) of these patients, the necessary data were available to determine the presence of sarcopenia and myosteatosis. Patients for whom height data or a (suitable) CT scan were not available were excluded from further analysis. Analysis of included and excluded patients revealed significantly more type-B injuries in the excluded group (*p* = 0.03). There were no differences in patient characteristics between the groups. For sarcopenia, the calculated sex-specific cut-off values were 47.7 cm^2^/m^2^ for men and 34.1 cm^2^/m^2^ for women. For myosteatosis, the calculated BMI-specific cut-off values were 26.2 mean HU for BMI < 25 kg/m^2^ and 25.9 mean HU for BMI ≥ 25 kg/m^2^. Patient characteristics are presented in Table 1. The reference group consisted of patients without sarcopenia and myosteatosis. Eventually, 66 patients (33%) were diagnosed with sarcopenia and 65 (32%) with myosteatosis. When dividing the groups, there were 98 patients (49%) without sarcopenia and myosteatosis (reference group), 36 patients (18%) with sarcopenia but without myosteatosis, 35 (18%) with myosteatosis but without sarcopenia and 30 (15%) with both (Figure 4). Compared to the reference group, sarcopenic patients had a higher age at the time of injury and more often had suffered a high-energy trauma. Patients with myosteatosis differed from the reference group in terms of more females and higher age at the time of injury. Patients with both sarcopenia and myosteatosis differed from the reference group in all characteristics except for treatment method and complication rates (Table 1).

### 3.2. Mortality and Survival

**Figure 5:**
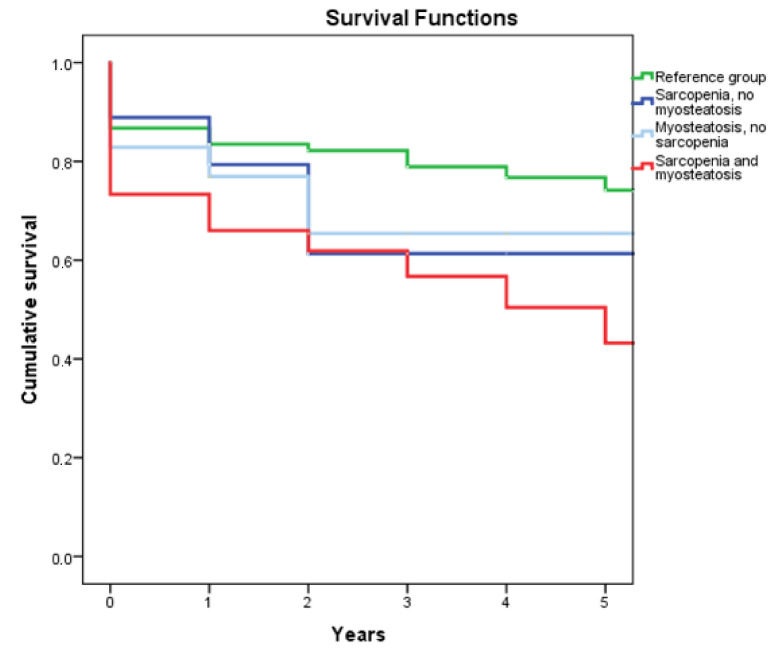
Kaplan–Meier survival curve of patients with or without sarcopenia and/or myosteatosis within the first five years post-injury. The green line represents the reference group (patients without sarcopenia or myosteatosis).

Analysis of mortality rates at different timepoints revealed that patients with sarcopenia had an increased mortality risk three years after the injury (Table 2). Patients with sarcopenia and myosteatosis had an increased mortality risk overall. Five out of thirteen patients (38%) who died within thirty days had myosteatosis or both sarcopenia and myosteatosis. One-, three- and five-year mortality rates were respectively 18 out of 31 (58%), 37 out of 58 (64%) and 42 out of 73 (58%) patients. Survival analysis revealed that patients who suffered from both sarcopenia and myosteatosis had the lowest survival rates, with over 50% mortality within the first five years post-injury (Figure 5). This was almost equally followed by patients with sarcopenia but not myosteatosis and patients with myosteatosis but not sarcopenia. Patients from the reference group showed the best long-term survival. In the univariable Cox proportional hazard analysis, sarcopenia and myosteatosis were not associated with overall survival (Table 3). Factors associated with survival in the multivariable analysis were age at the time of injury and CCI. An additional Cox regression analysis including only patients without intravenous contrast CT yielded similar results, with only age ≥ 86 years being associated with survival (HR 1.69, 95% CI 2.21–13.49, *p* =< 0.001).

### 3.3. Physical Functioning and Quality of Life in Patients with Sarcopenia and Myosteatosis

The results of the SMFA and EQ-5D are presented in Table 4. Out of 120 eligible patients for follow-up by means of PROMs, 90 patients (75%) responded (Figure 2) at a mean follow-up of 2.4 ± 2.2 years. The other 30 patients did not want to participate or were unable due to cognitive dysfunction. A non-response analysis revealed no differences between respondents and non-respondents. Out of the 90 respondents, 16 (18%) had sarcopenia or myosteatosis and 7 (8%) had both. Multivariable linear regression analyses were conducted to investigate whether the presence of sarcopenia and/or myosteatosis was associated with the level of physical functioning or QoL (Table 5). A significant decrease was found on the mental and emotional problems subscale of the SMFA in patients with sarcopenia. No other significant relation between sarcopenia or myosteatosis and patient-reported outcomes could be established for patients who were still alive and responded at a mean follow-up of 2.4 ± 2.2 years. An additional multivariable linear regression analysis including only patients without intravenous contrast CT yielded no relation between sarcopenia or myosteatosis and patient-reported outcomes (Supplementary Table S1).

## 4. Discussion

The present study assessed the prevalence of sarcopenia and myosteatosis in elderly patients with a pelvic ring injury and their influence on mortality as well as patient-reported physical functioning and QoL. In our study cohort, 33% of patients suffered from sarcopenia, 32% from myosteatosis and 15% of them had both. Patients with sarcopenia showed higher mortality rates after three years compared to non-sarcopenic patients. Survival in the first five years post-injury was lowest in patients with both sarcopenia and myosteatosis. Higher age and more comorbidities were independent risk factors for mortality, while sarcopenia and/or myosteatosis were not. In the patients still alive and responding after the two-year follow-up, no other relation with patient-reported physical functioning and QoL was established besides increased mental and emotional problems in patients with sarcopenia. This might be explained by fact that most survivors did not have sarcopenia or myosteatosis. 

Analysis revealed that 33% of our population dealt with sarcopenia. To the best of our knowledge, no other studies have evaluated rates of sarcopenia and myosteatosis and their relation to mortality in patients with pelvic ring injuries. General prevalence of sarcopenia was shown to be 1–33% across different populations [25]. When comparing the presence of sarcopenia in our population to populations with fractures in other body parts, some similar numbers were found. Iolascon et al. found that 23% of female patients aged > 55 with a single vertebral fracture had sarcopenia [26]. In contrast, Hida et al. found that 47% of female patients aged > 55 with a hip fracture dealt with sarcopenia, as compared to 81% of male patients [27]. Their estimation of muscle mass could be affected by surgical intervention and disuse atrophy, with the possibility to overestimate the prevalence. In our study, 32% of patients suffered from myosteatosis, similarly to the rates found by Vedder et al. [28] in patients with peripheral arterial occlusive disease (38%) and O’brien et al. [29] in patients with inflammatory bowel disease (34%). 

Analysis of mortality rates at different timepoints revealed that patients with sarcopenia had an increased mortality risk three years post-injury. More than half of patients that passed away after one, three and five years suffered from sarcopenia and/or myosteatosis. Survival was the worst in patients suffering from both conditions. However, neither sarcopenia nor myosteatosis were shown to be independent risk factors for mortality. In a study by Mitchell et al. on acetabular fractures, sarcopenia in patients over age 60 was considered an independent risk factor for one-year mortality [30]. They measured sarcopenia with the psoas:lumbar vertebral index (PLVI) and considered patients in the lowest quartile as being sarcopenic. These factors could explain the differences. 

Numerous studies reveal a significant decrease in physical functioning and quality of life after pelvic ring injuries compared to population standards, regardless of the presence of sarcopenia or myosteatosis [11,31,32]. We found that sarcopenia was negatively correlated with the mental and emotional status of the patient. No other statistically significant negative effects on patient-reported outcomes and QoL were found in patients with sarcopenia, myosteatosis or both. A possible explanation could be the relatively small number of patients per group, or that patients with a severely declined physical condition had already passed away by the time this cross-sectional study was conducted. Patients alive at least one-year post-injury were invited to participate, with a mean follow-up of 2.4 ± 2.2 years, so patients with a worse physical condition would likely have died before they could have been invited to participate in this study (survivorship bias). Several other studies found that sarcopenia was related to decreased physical functioning. Baumgartner et al. found that sarcopenia was significantly associated with self-reported physical disability in a large general population of community-dwelling men and women, independently of ethnicity, age, morbidity, obesity or income [2]. Patel et al. [33] found lower self-reported general health and physical functioning as measured by the SF-36 questionnaire in a general elderly population with sarcopenia, compared to non-sarcopenic age peers. The systematic review of Beaudart et al. [34] on QoL in various diseased and healthy sarcopenic populations revealed heterogeneous outcomes. Some studies found no difference in QoL between sarcopenic and non-sarcopenic participants, while others showed poorer QoL for sarcopenic patients or only poorer results in specific QoL domains. No studies were found evaluating patient-reported physical functioning and/or QoL in patients with myosteatosis. 

With sarcopenia and myosteatosis being common clinical problems in the frail elderly together with the high mortality rates shown in this study, some general recommendations for clinical practice can be made. Physicians should be aware that routine CT scans—initially performed for pelvic ring injury assessment—also contain valuable information about the presence of sarcopenia and/or myosteatosis. As these patients are prone to high mortality rates, multidisciplinary treatment should be considered, that includes consultation with a physiotherapist and dietician. The combination of various types of exercises, particularly resistance training, may improve muscle strength and physical performance if performed for at least three months [25]. No consistent effect of protein supplementation has been established [25], but essential amino acids (with leucine) and β-hydroxyβ-methylbutyric acid (HMB) proved to have some positive effects on muscle mass and muscle function. 

We believe to have addressed several clinically important issues. This is the first study to provide insight into the prevalence of sarcopenia and myosteatosis in elderly patients with a pelvic ring injury. It is also the first to establish a possible relation between sarcopenia, myosteatosis and mortality, as well as between sarcopenia, myosteatosis and patient-reported physical functioning and QoL. We had a high response rate of 75% on the PROMs, despite this being a fragile population.

When interpreting the results of our study, some limitations should also be taken into account. Suboptimal positioning of the patient in the CT scan in the acute setting could have caused some imaging artefacts, possibly influencing sarcopenia and myosteatosis measurements. In some cases, intravenous contrast was used, which can increase radiodensity [24] and thus lower the reliability of myosteatosis measurements. We therefore performed additional Cox regression and multivariable analyses of the 61 CT scans with intravenous contrast that yielded similar results. Muscle measurements are typically taken at the level of the third lumbar vertebra (L3), as the cross-sectional skeletal muscle area at this level is highly correlated with total body skeletal muscle mass [35]. However, several studies revealed that L4 is a good alternative [35,36]. In the present study, many patients were excluded from further analysis, as L4 was not always included in routine CT scans of the pelvis. Still, analysis of included and excluded patients based on the availability of usable CT scans yielded no differences in patient characteristics. Although interest in sarcopenia and myosteatosis is growing considerably, widely accepted definitions and adequate cut-off values suitable for use in research are still lacking. So far, no fixed criteria exist for identifying the level at which relative muscle mass becomes deficient. The European Working Group on Sarcopenia in Older People (EWGSOP) recommends cut-off values set at two standard deviations below the mean of a healthy, young adult population [37]. In addition to gait speed for performance and handgrip testing for strength, gold standards for measurement of sarcopenia include computed tomography (CT) for muscle mass. In the absence of functional testing data, which was the case in our study due to its retrospective nature, sarcopenia assessment could be completed from CT alone. However, measurement techniques for sarcopenia vary widely and also include thresholds based on measurements derived from DXA scans and cut-off points based on optimal stratification methods. No cut-off values are available in the literature for this population, as no previous studies exist that assessed rates of sarcopenia and myosteatosis in a population with pelvic ring injuries and measured at the level of L4. Hence, we used the lowest tertiles as cut-off points, which is common in the assessment of sarcopenia and myosteatosis [38,39]. They provide reliable values based on a specific population. Third and most importantly, the retrospective nature of this study makes it prone to survivorship bias or survival bias. This is a form of selection bias that results from the focus on survivors instead of a broader context that includes those that did not survive. This may lead to a distorted and possibly overly optimistic image of the results. In our cohort, thirty-one (16%) of the patients died within one year after the pelvic ring injury and could therefore not be included for follow-up analysis with PROMs, and 18 (58%) of them had sarcopenia, myosteatosis or both. This could be a feasible explanation for the fact that, besides the relation with mental and emotional problems, no other statistically significant association could be established between sarcopenia and/or myosteatosis and physical functioning and QoL.

## 5. Conclusions

About half of patients over 65 years of age with a pelvic ring injury had sarcopenia, myosteatosis or both. Mortality in the first few years after the injury was high among patients with sarcopenia and/or myosteatosis compared to patients without these conditions. There was a negative correlation between sarcopenia and patients’ mental and emotional status. No other statistically significant differences could be highlighted between the presence of sarcopenia and/or myosteatosis and patient-reported physical functioning and QoL at long-term follow-up. Further prospective studies on larger groups of patients are necessary to evaluate whether sarcopenia and/or myosteatosis are potential predictive factors for decreased physical functioning and QoL in elderly patients with a pelvic ring injury, as well as intervention studies for the effects of muscle training and dietary adaptations.

## Figures and Tables

**Table 1:** Baseline characteristics of patients with a pelvic ring injury.

	All Patients (*N* = 199)	Reference Group ^†^ (*N* = 98)	Sarcopenia (*N* = 36)	*p*-Value ^‡^	Myosteatosis (*N* = 35)	*p*-Value ^‡^	Sarcopenia + Myosteatosis (*N* = 30)	*p*-Value ^‡^
Gender *				0.44		**0.04**		**0.05**
Male	70 (35)	39 (40)	17 (47)		8 (23)		6 (20)	
Female	129 (65)	59 (60)	19 (53)		27 (77)		24 (80)	
**Age at injury** (mean ± SD)	78 ± 8	75 ± 7	79 ± 8	**0.03**	79 ± 8	**0.05**	83 ± 7	**<0.001**
**BMI** (kg/m^2^) (mean ± SD)	25 ± 5	25 ± 4	24 ± 4	0.08	27 ± 6	0.09	24 ± 4	**0.05**
BMI < 25	22 (11)	50 (51)	21 (59)		13 (37)		22 (73)	
BMI ≥ 30	177 (89)	48 (49)	15 (42)		22 (63)		8 (27)	
**SMI** (cm^2^/m^2^) (mean ± SD)	41.6 ± 9.3	46.1 ± 8.5	36.7 ± 6.9	**<0.001**	42.5 ± 8.2	0.46	32.0 ± 4.9	**<0.001**
Male	49.9 ± 7.2	53.8 ± 4.7	43.2 ± 2.9		53.3 ± 6.4		38.5 ± 5.6	
Female	37.2 ± 7.0	41.0 ± 6.3	30.8 ± 2.7		39.3 ± 5.4		30.5 ± 3.2	
**Mean muscle HU** (mean ± SD)	31.1 ± 10.3	37.3 ± 7.5	34.7 ± 6.7	**0.06**	21.2 ± 3.7	**<0.001**	18.2 ± 5.3	**<0.001**
**Sarcopenia**	-	-	36 (100)		-	-	30 (100)	
**Myosteatosis**	76 (38)	-	-	-	35 (100)		30 (100)	
**CCI** (mean ± SD)	6 ± 2	5 ± 2	5 ± 2	0.41	5 ± 2	0.13	6 ± 2	**<0.001**
**Injury mechanism**				**0.04**		0.38		**<0.001**
Low-energy trauma	122 (61)	48 (49)	25 (69)		22 (63)		27 (90)	
High-energy trauma	77 (39)	50 (51)	11 (31)		13 (37)		3 (10)	
**ISS** (mean ± SD)	14 ± 11	15 ± 12	11 ± 9	0.06	16 ± 12	0.65	10 ± 9	**0.03**
**Injury classification**				0.21		0.41		**0.007**
Type A	68 (34)	25 (26)	13 (36)		14 (40)		16 (53)	
Type B	113 (57)	61 (62)	20 (56)		20 (57)		12 (40)	
Type C	18 (9)	12 (12)	3 (8)		1 (3)		2 (7)	
**Treatment**				0.46		0.77		0.24
Non-operative	165 (83)	79 (81)	31 (86)		28 (80)		27 (90)	
Operative	34 (17)	19 (19)	5 (14)		7 (20)		3 (10)	
**Complications ≤ 30 days**	58 (30)	28 (29)	9 (25)	0.58	13 (37)	0.33	8 (27)	0.74

* Numbers are expressed as N (%) unless otherwise specified. ^†^ Reference group: all patients without sarcopenia and/or myosteatosis. ^‡^ Patients with or without sarcopenia and/or myosteatosis were compared with the reference group. Statistically significant results are presented in bold. BMI: body mass index, SMI: skeletal muscle index, HU: Hounsfield units, CCI: Charlson Comorbidity Index, ISS: injury severity score.

**Table 2:** Mortality analysis of patients with and without sarcopenia and/or myosteatosis.

	All Patients (*N* = 199)	Reference Group * (*N* = 98)	Sarcopenia (*N* = 36)	*p*-Value	Myosteatosis (*N* = 35)	*p*-Value	Sarcopenia + Myosteatosis (*N* = 30)	*p*-Value
Deceased, N (%)	59 (30)	26 (27)	12 (33)	0.44	12 (34)	0.49	16 (53)	**0.006**
<30 days	13 (7)	8 (8)	0 (0)	0.55	2 (6)	0.55	3 (10)	0.81
<1 year	31 (16)	13 (13)	4 (11)	0.82	6 (17)	0.39	8 (27)	0.46
<3 years	58 (29)	21 (21)	13 (36)	**0.003**	11 (31)	0.21	13 (43)	0.23
<5 years	73 (37)	31 (32)	13 (36)	0.09	12 (34)	0.56	17 (57)	0.20

* Mortality rates of patients with only sarcopenia, only myosteatosis and both sarcopenia and myosteatosis were compared to the reference group of patients without sarcopenia or myosteatosis. Statistically significant results are in bold.

**Table 3:** Univariable and multivariable Cox proportional hazards model for overall survival.

	Univariable Analysis			Multivariable Analysis		
	HR	95% CI	*p*-Value	HR	95% CI	*p*-Value
**Sex**						
Male	Ref					
Female	0.33	0.78–2.47	0.27			
**Age**						
65–75	Ref					
76–85	1.02	1.41–5.42	**0.003**	1.09	1.54–5.74	**0.001**
≥86	1.51	2.18–9.47	**<0.001**	1.65	2.55–10.56	**<0.001**
**Sarcopenia/myosteatosis**						
No sarcopenia, no myosteatosis	Ref					
Sarcopenia, no myosteatosis	0.36	0.70–2.91	0.33			
Myosteatosis, no sarcopenia	−0.05	0.46–1.95	0.89			
Sarcopenia and myosteatosis	0.35	0.73–2.76	0.31			
**Charlson Comorbidity Index**						
2–3	Ref					
≥4	0.56	1.01–3.07	**0.05**	0.60	1.07–3.12	**0.03**
**Injury Severity Score**						
0–16	Ref					
≥16	0.30	0.77–2.39	0.29			

HR: hazard ratio, Ref: reference category, CI: confidence interval.

**Table 4:** Patient-reported physical functioning and QoL in patients with sarcopenia and myosteatosis.

	Reference Group (*N* = 51)	Sarcopenia (*N* = 16)	Myosteatosis (*N* = 16)	Sarcopenia + Myosteatosis (*N* = 7)
SMFA *				
Function	77 (61–92)	88 (75–96)	75 (60–89)	74 (48–79)
Bother	79 (60–92)	90 (77–98)	79 (57–90)	71 (33–79)
LE	79 (52–94)	88 (76–97)	72 (61–87)	71 (48–79)
ADL	75 (50–89)	83 (67–98)	75 (51–87)	65 (34–71)
Emotion	78 (63–88)	91 (80–94)	78 (67–90)	75 (72–88)
EQ-5D *	0.69 (0.31–1.00)	0.78 (0.23–1.00)	0.75 (0.39–0.88)	0.66 (0.37–0.78)

* Expressed as median (IQR). IQR: interquartile range, ADL: activities of daily living, LE: lower extremity.

**Table 5:** Multivariable linear regression analysis.

	Group *	B	95% CI	*p*-Value
**SMFA**				
Function	Sarcopenia ^†^	10.25	−1.68, 22.18	0.09
	Myosteatosis ^‡^	1.53	−9.49, 12.55	0.78
	Sarcopenia + myosteatosis ^§^	−3.73	−21.97, 14.52	0.68
Bother	Sarcopenia	11.15	−1.61, 23.91	0.09
	Myosteatosis	0.97	−10.99, 12.93	0.87
	Sarcopenia + myosteatosis	−10.87	−30.66, 8.91	0.28
LE	Sarcopenia	10.67	−2.49, 23.84	0.11
	Myosteatosis	1.48	−10.69, 13.65	0.81
	Sarcopenia + myosteatosis	−2.78	−23.62, 16.06	0.70
ADL	Sarcopenia	12.30	−1.69, 26.25	0.08
	Myosteatosis	2.13	−10.83, 15.09	0.74
	Sarcopenia + myosteatosis	−8.74	−30.21, 12.73	0.42
Emotion	Sarcopenia	10.61	−0.04, 21.26	**0.05**
	Myosteatosis	1.72	−8.19, 11.62	0.73
	Sarcopenia + myosteatosis	0.82	−15.39, 17.04	0.92
**EQ-5D**	Sarcopenia	−0.03	−0.23, 0.18	0.81
	Myosteatosis	0.008	−0.18, 0.19	0.93
	Sarcopenia + myosteatosis	−0.07	−0.35, 0.21	0.61

* Group without sarcopenia or myosteatosis is the reference group. ^†^ Corrected for CCI and BMI. ^‡^ Corrected for CCI and age. ^§^ Corrected for CCI, BMI and age. ADL: activities of daily living, LE: lower extremity.

## Data Availability

The data presented in this study are available on request from the corresponding author. The data are not publicly available due to privacy restrictions.

## Supplementary Materials

The following are available online at https://www.mdpi.com/article/10.3390/jcm10214874/s1, Table S1: multivariable linear regression analysis of patients without intravenous contrast CT.
