# Supplementary material for: Are Sarcopenia and Myosteatosis in Elderly Patients with Pelvic Ring Injury Related to Mortality, Physical Functioning and Quality of Life?
Source: J Clin Med. 2021 Oct 22;10(21):4874. doi: 10.3390/jcm10214874 (PMC8584633; doi:10.3390/jcm10214874)
Supplement: Supplementary file 1 [file jcm-10-04874-s001.zip › jcm-1414522-Supplementary.pdf]

Table S1. multivariable linear regression analysis of patients without intravenous contrast CT.

	Group*	B	95% CI	*p*-value
**SMFA**				
Function	Sarcopenia**	5.79	−9.32, 20.89	0.44
	Myosteatosis†	4.52	−8.20, 17.23	0.48
	Sarcopenia + myosteatosis₸	−10.99	−31.54, 9.55	0.28
Bother	Sarcopenia	5.13	−9.73, 19.98	0.49
	Myosteatosis	0.70	−12.24, 13,65	0.91
	Sarcopenia + myosteatosis	−19.51	−40.09, 1.08	0.06
LE	Sarcopenia	4.60	−11.93, 21.13	0.58
	Myosteatosis	5.36	−8.54, 19,26	0.44
	Sarcopenia + myosteatosis	−13.25	−35.09, 8.59	0.23
ADL	Sarcopenia	6.78	−10.26, 23.82	0.43
	Myosteatosis	3.95	−10.49, 18.39	0.59
	Sarcopenia + myosteatosis	−16.96	−40.39, 6.38	0.15
Emotion	Sarcopenia	7.03	−4.83, 18.89	0.24
	Myosteatosis	−0.22	−10.93, 10.49	0.97
	Sarcopenia + myosteatosis	−5.14	−21.41, 11.14	0.53
**EQ-5D**	Sarcopenia	−0.06	−0.33, 0.21	0.64
	Myosteatosis	0.05	−0.19, 0.29	0.68
	Sarcopenia + myosteatosis	−0.15	−0.49, 0.20	0.39

*Group without sarcopenia or myosteatosis is the reference group; ** corrected for CCI and BMI; † corrected for CCI and age; ₸ corrected for CCI, BMI and age; ADL activities of daily living; LE lower extremity.
